# The Association between Hypoperfused Myocardium and Systolic Function in Patients With AMI Undergoing Complete Revascularization

**DOI:** 10.1002/clc.70437

**Published:** 2026-08-06

**Authors:** Jin Si, Chong Zheng, Jinghao Sun, Keling Xiao, Yadong Cui, Bixiao Cui, Ming Yi, Lijie Sun, Haoyu Zhang, Shanshan Gu, Jie Lu, Jing Li

**Affiliations:** ^1^ Department of Geriatrics, Xuanwu Hospital Capital Medical University National Clinical Research Center for Geriatric Disease Beijing China; ^2^ Department of Radiology and Nuclear Medicine Xuanwu Hospital Capital Medical University Beijing China

**Keywords:** acute myocardial infarction, hypoperfused myocardium, PET/MR, revascularization

## Abstract

**Background:**

Timely restoring coronary blood flow reduces mortality in acute myocardial infarction (AMI), while epicardial flow restoration is non‐parallel to myocardial reperfusion. This prospective study examined temporal changes in myocardial perfusion and their association with left ventricular (LV) systolic function after complete revascularization.

**Methods:**

AMI patients treated with complete revascularization underwent hybrid positron emission tomography/magnetic resonance imaging (PET/MR) at 2 months and 1 year after the index procedure. Hypoperfused myocardium was defined as reduced perfusion with preserved metabolism.

**Results:**

Fifty‐five patients (60.42 ± 11.47 years, 80.0% male) were enrolled, and hypoperfused myocardium (median: 12% of LV) was detected in 25 (45.5%) patients 2 months post‐AMI. Patients with and without hypoperfused myocardium were classified as Group A and Group B, respectively. Higher high‐sensitivity C‐reactive protein (hs‐CRP) and lower T2 values were observed in Group A and independently predicted baseline hypoperfused myocardium. At 1‐year PET/MR follow‐up, six (31.6%) patients in Group A showed perfusion recovery; these patients were more frequently female (*p* = 0.025), less frequently diabetic (*p* = 0.044) or overweight (*p* = 0.035), and had better baseline global longitudinal strain (GLS) (*p* = 0.005). Significant LV ejection fraction improvement was observed in Group A (56.39% ± 9.76% vs. 54.58% ± 10.39%, *p* = 0.016) but not in Group B (51.63% ± 13.09% vs. 50.79% ± 16.95%, *p* = 0.615).

**Conclusions:**

Even after complete revascularization, myocardial hypoperfusion may persistently exist and evolve over time in AMI patients. The reversal of myocardial hypoperfusion is possibly associated with improved LV systolic function.

**Trial Registration:** The study was retrospectively registered on 2022‐11‐24 under the name Evaluation of short—and long‐term cardiac structure and function in patients with acute coronary syndrome and made publicly available in the China Medical Research Registration Information System (https://www.medicalresearch.org.cn/), with the registration number MR.‐11‐23‐000090.

## Graphical abstract

Hypoperfused myocardium was detected in portions of acute myocardial infarction patients undergoing complete revascularization by hybrid positron emission tomography/magnetic resonance at 2 months. Patients with hypoperfused myocardium (Group A) had higher hs‐CRP and lower T2 values than those without (Group B). The reversal of myocardial perfusion was observed in some patients at 1 year, and may be associated with improvements in systolic function.

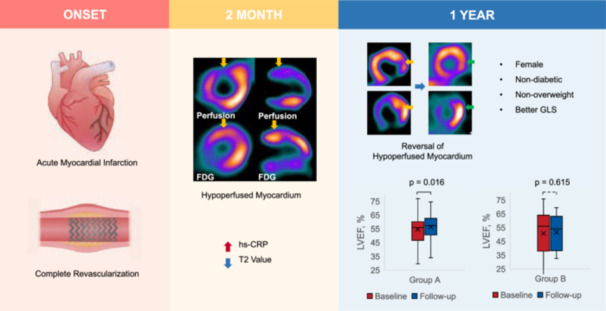

**Abbreviations**
AMI: acute myocardial infarction
FDG: fluorodeoxyglucose
GLS: global longitudinal strain
hs‐CRP: high‐sensitivity C‐reactive protein
LGE: late gadolinium enhancement
LVEF: left ventricular ejection fraction
PCI: percutaneous coronary intervention
PET/MR: positron emission tomography/magnetic resonance

## 1 Introduction

Acute myocardial infarction (AMI) is one of the leading causes of cardiovascular death worldwide [1]. Timely reperfusion is a proven strategy to salvage ischemic myocardium, improve outcomes, and is recommended in guidelines as the standard treatment strategy [2]. Opening the vessel is generally considered a sign of successful revascularization, while patency of the epicardial coronary artery is not equivalent to reperfusion of the myocardium. Despite the restoration of Thrombolysis In Myocardial Infarction (TIMI) grade 3 flow, myocardial perfusion, as evidenced by myocardial blush, was normal in only 29% of the AMI patients following primary percutaneous coronary intervention (PPCI) [3]. Likewise, reduced or absent reperfusion of myocardium was detected by contrast echocardiography in over 80% of ST‐segment elevation myocardial infarction (STEMI) patients within 48 h of successful PPCI [4]. Furthermore, a cardiac magnetic resonance (CMR) study also showed abnormal myocardial perfusion in 54% of the patients 2 days post reperfused AMI [5]. Underlying mechanisms consist of myocyte swelling, endothelial injury, microembolization, and inflammatory cell infiltration [6]. The aforementioned studies revealed the phenomenon of myocardial hypoperfusion post‐AMI, but the dynamic of hypoperfused myocardium and its association with left ventricular (LV) systolic function has yet to be comprehensively studied. Besides, it is noteworthy that previous studies were conducted through various image modalities based on different physiological foundations of the myocardium, which might lead to obscurity in integration and interpretation.

F‐18 fluorodeoxyglucose (FDG) positron emission tomography (PET) is the gold standard for identifying reversible myocardial dysfunction [7], and the underlying principle is that ischemia leads to a shift in myocardial metabolism from the oxidation of free fatty acids to glucose utilization [8]. Late gadolinium enhancement (LGE) CMR accurately estimates scars as signal‐enhanced areas and serves as the standard. Hybrid positron emission tomography/magnetic resonance (PET/MR) combines the high‐resolution morphological and functional assessment afforded by CMR with the ability of PET to quantify perfusion and metabolism, and therefore provide incremental diagnostic value.

In this study, AMI patients were imaged at 2 months and 1 year after complete revascularization using hybrid PET/MR for myocardial perfusion, metabolism, and LV systolic function assessment. We aimed to verify our hypothesis that hypoperfused myocardium is dynamically changing and may be associated with LV systolic function.

## 2 Patients and Methods

### 2.1 Study Population

This investigator‐initiated study prospectively enrolled patients admitted as AMI and underwent complete revascularization from October 2020 to July 2021 at Xuanwu Hospital Capital Medical University. Inclusion criteria were as follows [1]: ≥ 18 years old [2]; definitive AMI diagnosis as per current guidelines [9], consisting of STEMI and non‐ST‐segment elevation myocardial infarction (NSTEMI), and [3] complete revascularization of the epicardial coronary artery. Patients were excluded for [1] New York Heart Association (NYHA) Ⅳ [2]; estimated glomerular filtration rate (eGFR) < 30 ml/min/1.73m^2^ [3]; allergy to contrast [4]; claustrophobia [5]; ferromagnetic material implantation [6]; pregnancy [7]; life expectancy ≤ 1 year due to non‐cardiac complication [8]; patient's refusal. The baseline hybrid PET/MR scan was scheduled 2 months after admission. All patients were treated with optimal medical therapy according to contemporary guidelines [10, 11]. Patients' information (clinical characteristics, laboratory results, and angiography) was collected by inquiry and reviewing medical records.

This study was performed in line with the principles of the Declaration of Helsinki. Approval was granted by the Ethics Committee of Xuanwu Hospital Capital Medical University (Clinical trial [2022] No.169), and written informed consent was obtained from all patients. The corresponding author has full access to all the data in the study and takes responsibility for its integrity and data analysis.

### 2.2 Biochemical Measurements

Peripheral blood samples were obtained within 24 h of hospital admission, blood cell counts and serum concentrations of creatinine, uric acid, hemoglobin, glycosylated hemoglobin (HbA1c), total cholesterol, and low‐density lipoprotein‐cholesterol (LDL‐C), high‐sensitivity C‐reactive protein (hs‐CRP) and interleukin‐6 (IL‐6) were assessed by standard laboratory methods. creatine kinase‐MB (CKMB) and cardiac troponin I (cTnI) were tested every 6 h within the first 24 h after admission, and the highest values were taken into further analysis.

### 2.3 Revascularization

Diagnostic coronary angiography was performed using 5 to 6 French Judkins catheters (Cordis Corp., Miami Lakes, Florida, USA) through either a radial or femoral approach. Single‐vessel disease was defined as stenosis of at least 50% in one major epicardial coronary artery, while multi‐vessel disease was defined as stenosis of at least 50% in ≥ two major epicardial coronary arteries. STEMI patients received PPCI, and residual stenosis of the non‐culprit coronary artery was subsequently managed in elective procedures. The timing of the invasive strategy for NSTEMI patients was determined according to risk identification. Revascularization strategy, percutaneous coronary intervention (PCI), or coronary artery bypass graft (CABG) was under the operators' discretion. All patients received complete revascularization of epicardial coronary.

### 2.4 PET/MR Acquisition

Cardiac PET and CMR were performed in all patients using a hybrid 3.0 T PET/MR scanner (uPMR790, Unite Imaging Healthcare, Shanghai, China) that allows the simultaneous acquisition of PET and CMR data in a single examination.

### 2.5 PET Imaging

Assessment of myocardial viability using ^13^N‐NH_3_·H_2_O resting perfusion imaging and ^18^F‐FDG PET imaging. PET was performed after a fasting period of at least 12 h. The 20 min Dynamic 3D list mode imaging began immediately after intravenous injection of 529.25 ± 121.08 MBq ^13^N‐NH_3_·H_2_O and comprised an electrocardiography‐gated cardiac PET acquisition with ordered subset expectation maximization (OSEM) image reconstruction (voxel size, 3 × 3 × 3 mm) with ordinary Poisson ordered‐subset expectation maximization with two iterations and 20 subsets, a Gaussian filter with 5.0‐mm full width at one‐half maximum, and a 150 × 150 matrix size.

Within 1 day of ^13^N‐NH_3_·H_2_O resting perfusion imaging, ^18^F‐FDG PET imaging was performed to assess myocardial metabolism. According to the American Society of Nuclear Cardiology PET imaging guidelines [12], blood glucose levels were controlled by oral glucose loading and, if necessary, by supplemental intravenous insulin. Intravenous injection of ^18^F‐FDG 3.7 MBq/kg was given when the blood glucose concentration was within 5.5−7.7 mmol/L. A four‐compartment model attenuation map was calculated from fat‐ and water‐only Dixon‐based sequences by segmentation into background, lung, fat, and soft tissues for automatic attenuation correction of the acquired PET data.

### 2.6 CMR Imaging

A 12‐channel cardiac phase‐array coil was used for the entire scan, and images were acquired in end‐expiratory breath‐hold under electrocardiographic gating. A balanced steady‐state free precession (bSSFP) sequence was used to acquire cine images of two‐chamber, four‐chamber, and short‐axis slices with the following parameters: repetition time (TR)/echo time (TE), 3.1/1.4 ms; field of view (FOV), 360 × 320 mm^2^; matrix, 192 × 192; slice thickness/gap, 8/2 mm; flip angle (FA), 40°; acceleration factor, 2; 25 phases per cardiac cycle. Eight to twelve contiguous short‐axis slices were applied to cover the entire left ventricle. To assess LGE, phase‐sensitive, inversion‐recovery (PSIR) prepared T1‐weighted gradient‐echo pulse sequence was acquired 10‐15 min after the intravenous injection of 0.15 mmol/kg Magnevist (Bayer Schering Pharma, Berlin, Germany). Parameters are as follows: TR/TE, 4.7/1.9 ms; FOV 360 × 360 mm^2^; matrix, 240 × 180; slice thickness/gap, 8/2 mm; FA, 20°; acceleration factor, 2; TI, 300−330 ms. A 3(3)3(3)5 scheme modified Look‐Locker inversion recovery (MOLLI) sequence was used for T1 mapping before and after contrast administration in the short‐axis plane at the basal, middle, and apical LV. The starting TI was selected as 100 ms, with an increment of 80 ms for the subsequent look‐locker trains. Parameters are as follows: TR/TE, 2.9/1.3 ms; FA, 35^°^; FOV, 360 × 320 mm^2^; matrix, 256 × 159; slice thickness/gap, 8/2 mm. T2 mapping was performed using a T2‐prepared steady‐state free precession (T2p‐SSFP) sequence with the same slice location as T1 mapping with the following parameters: TR/TE, 3.0/1.4 ms; preparation times, 0 ms, 30 ms, 55 ms; FA, 35^°^; FOV, 360 × 320 mm^2^; matrix, 288 × 229; slice thickness/gap, 8/2 mm.

### 2.7 PET/MR Analysis

PET/MR images were analyzed by two experienced radiologists according to the American Heart Association (AHA) 17‐segment model [13]. Hypoperfused myocardium was identified and quantified based on a consensus reading of the two observers. Any discrepancies in image interpretation were resolved by discussion.

### 2.8 CMR Analysis

CMR images were analyzed by using CVI 42 (Circle Cardiovascular Imaging Inc., Client Version 5.14.2, Calgary, Alberta, Canada). The endocardial and epicardial contours at the end‐systolic and end‐diastolic phases of the left ventricle were outlined on the short‐axis cine images to estimate LV volume and function (end‐systolic volume, ESV; end‐diastolic volume, EDV; ejection fraction, EF; and LV myocardial mass, LVMM). Three‐dimensional global strain parameters (global longitudinal strain, GLS; global radial strain, GRS and global circumferential strain, GCS) were calculated by averaging peak values of all the segments. LGE was estimated by manually delineating the endocardial and epicardial contours in each slice of the short‐axis PSIR image. A hyperintense area with signal intensity ≥ 5 standard deviations (SD) than the remote normal region was considered as myocardial scar. The extent of LGE was summed as the total amount of scar in every short‐axis slice and expressed as both absolute value (in grams) and percentage of LVMM. Pixel‐wise native and post‐contrast T1 and T2 maps were generated from T1 mapping and T2 mapping images after motion correction. Global myocardial T1 and T2 values were manually estimated by outlining the LV myocardium contours, excluding the epicardial fat, pericardial effusion, and blood pool. ROIs in the blood pool were also drawn from T1 maps to estimate native and post‐contrast blood T1 values. Hematocrit (Hct) was obtained on the same day before the PET/MR scan to calculate the extracellular volume (ECV) based on the following equation: ECV = (1−Hct) × (ΔR1myocardium/ΔR1blood), where R1 = 1/T1 [14].

### 2.9 PET Analysis

PET image was analyzed using a medical image processing workstation (uWS, United Imaging Healthcare, Shanghai, China). Myocardial viability was determined according to segmental perfusion and metabolism. The hypoperfused segment was defined as ^13^N‐NH_3_·H_2_O uptake of less than 50% while ^18^F‐FDG uptake of no less than 50% of the remote region. Normal myocardium was defined as that with both normal ^13^N‐NH_3_·H_2_O and ^18^F‐FDG uptake, while myocardial scar was defined by ^13^N‐NH_3_·H_2_O and ^18^F‐FDG uptake less than 50% of the remote region [15]. The recovery of hypoperfused myocardium was defined as the recovery of perfusion in the index segment and thus resulted in the reduction of hypoperfused myocardium accompanied by an increased amount of normal myocardium. Absolute rest myocardial blood flow (MBF) was quantified through time‐activity curve data, fitted by a two‐compartment kinetic model corrected for spill‐over and partial volume effects, using Carimas software (version 2.10, Turku PET Center, Finland) [16]. MBF values were obtained based on coronary artery regions (LAD, LCX, and RCA) and expressed as mL/min/g.

### 2.10 Follow‐Up

All patients were followed up by contacts through telephone calls, outpatient visits, and review of the electronic medical records for clinical characteristics every 6 months. Follow‐up PET/MR scan and laboratory tests of blood cell counts, biochemical indexes and inflammatory indicators were scheduled 1 year after hospital admission.

### 2.11 Statistical Analysis

Continuous variables were expressed as mean ± SD if normally distributed or as median with interquartile range (IQR) if non‐normally distributed. Categorical variables were presented as frequencies and percentages. Between‐group comparisons of continuous variables were performed using Student's *t*‐test or the Mann–Whitney *U* test, as appropriate. Categorical variables were compared using Pearson's chi‐squared test or Fisher's exact test, as appropriate. For paired samples, paired‐samples *t*‐test or Wilcoxon signed‐rank test was used according to the distribution of the data.

Multivariable logistic regression analysis was performed to identify factors associated with the presence of hypoperfused myocardium. Results were presented as odds ratios (ORs) with 95% confidence intervals (CIs). AMI type was included as an adjustment variable in the multivariable model to account for potential differences between STEMI and NSTEMI patients. To further evaluate whether AMI type modified the associations between the main continuous variables and the outcome, T2 and hs‐CRP were mean‐centered, and interaction terms between AMI type and each centered continuous variable were generated. The interaction terms for AMI type × T2 and AMI type × hs‐CRP were simultaneously added to the multivariable logistic regression model. In addition, subgroup analyses stratified by AMI type were performed.

All tests were two‐sided, and a *p* value < 0.05 was considered statistically significant. Statistical analyses were performed using SPSS version 29.0 (IBM SPSS Statistics 29, IBM Corporation, Armonk, NY, USA).

## 3 Results

### 3.1 Study Population and Baseline Characteristics

**Figure 1:**
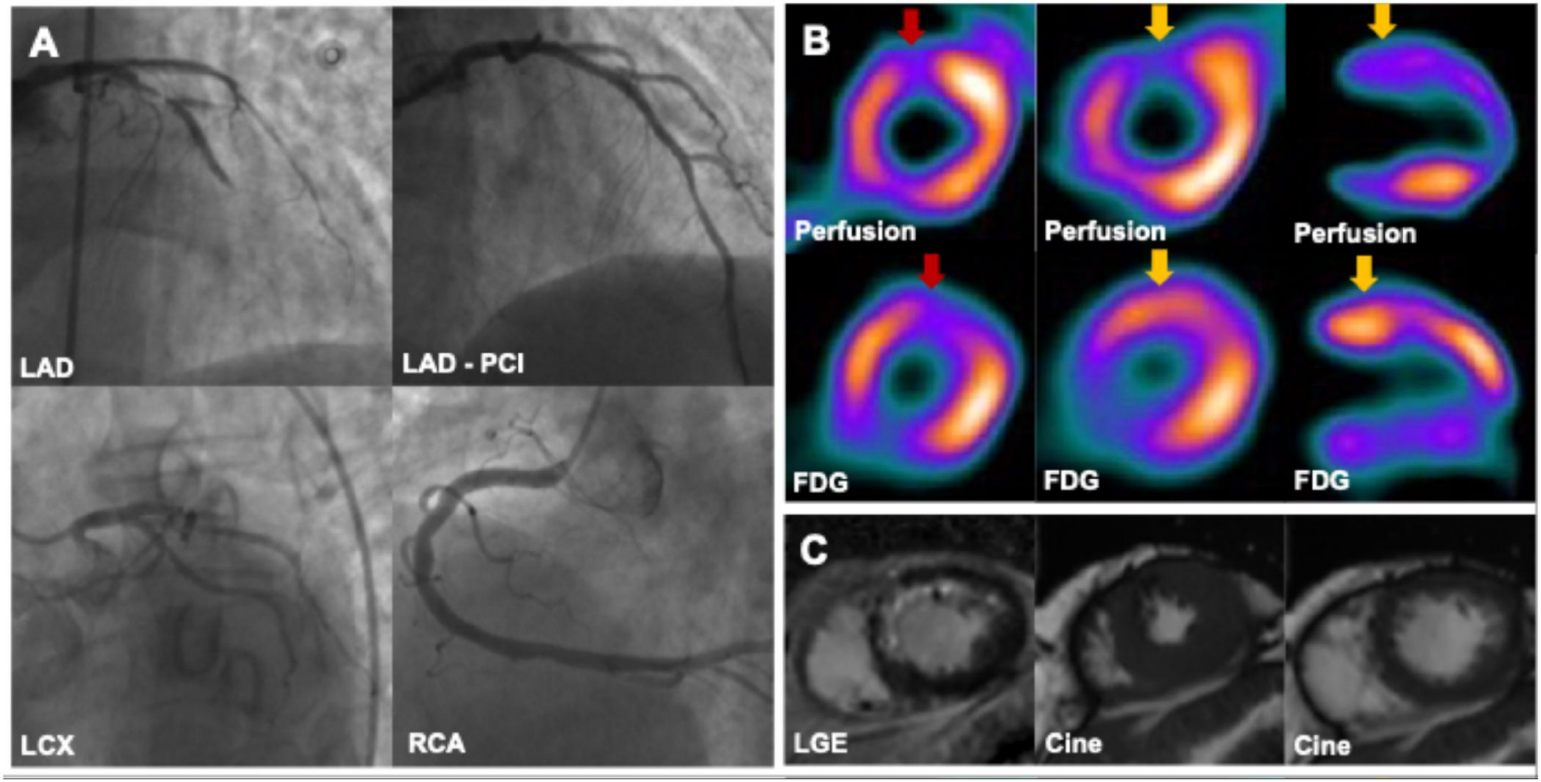
Hypoperfused myocardium in an AMI patient after complete revascularization. Multi‐modality image of a 69‐year‐old male initially presented with anterior STEMI. (A) Coronary angiography showing occlusion in the proximal LAD, subsequent stent implantation, and no significant stenosis in LCX or RCA. (B) PET showing hypoperfused myocardium (yellow arrow) in apical, partial mid, and basal anterior, and myocardial scar (red arrow) in partial mid anterior. C: CMR indicating scar in anterior (red arrow) and segmental wall movement abnormality in the region with hypoperfused myocardium. AMI = acute myocardial infarction; CMR = cardiac magnetic resonance; LAD = left anterior descending; LCX = left circumflex; LGE = late gadolinium enhancement; PCI = percutaneous coronary intervention; PET = positron emission tomography; RCA = right coronary artery; STEMI = ST‐segment elevation myocardial infarction.

Fifty‐five AMI patients, consisting of 28 STEMI and 27 NSTEMI, were enrolled in this study. There were 44 (80.0%) males and 11 (20.0%) females with a median age of 60.42 ± 11.47 years at study inclusion. The baseline hybrid PET/MR scan was performed 61 [30, 92] days after admission. Hypoperfused myocardium was detected in 25 (45.5%) patients, accompanied by segmental wall movement abnormality, localized in the perfusion territory of the culprit coronary artery (Figure 1).

### 3.2 Predictors of Baseline Hypoperfused Myocardium

For further comparison, patients were divided into Group A (with hypoperfused myocardium) and Group B (without hypoperfused myocardium). Patients in Group A had higher hs‐CRP levels [12.03 (6.16, 28.39) vs. 4.26 (2.26, 10.31), *p* = 0.020] and lower T2 value (51.87 ± 5.03 vs. 55.45 ± 4.54, *p* = 0.010) compared with Group B. No significant differences were observed in other demographic characteristics, clinical variables, biomarkers, or cardiac functional parameters between the two groups, as shown in Table 1.

**Table 1:** Baseline clinical characteristics according to the presence of hypoperfused myocardium.

Variable	Hypoperfused myocardium (+) (*n* = 25)	Hypoperfused myocardium (−) (*n* = 30)	*p* value
*Demographics*			
Age, years	60.36 ± 10.97	60.47 ± 12.07	0.973
Male, (*n*, %)	19 (76.0)	25 (83.3)	0.498
BMI, kg/m^2^	24.84 ± 2.60	25.48 ± 2.75	0.381
*Type of AMI*			
STEMI, *n* (%)	14 (56.0)	14 (46.7)	0.491
*Cardiovascular risk factors*			
Hypertension, (*n*, %)	15 (60.0)	17 (56.7)	0.803
Diabetes mellitus, (*n*, %)	11 (44.0)	11 (36.7)	0.580
Smoking, (*n*, %)	13 (52.0)	20 (66.7)	0.269
Overweight or obesity, (*n*, %)	19 (76.0)	21 (70.0)	0.619
*Timing of revascularization*			
STEMI			
Symptom to balloon, h	4.34 ± 2.20	4.19 ± 1.90	0.320
Door to balloon, min	29.64 ± 15.51	26.15 ± 13.35	0.560
NSTEMI			
Symptom to admission, day	1.50 (1.00, 2.00)	2.00 (0.96, 3.00)	0.923
Symptom to revascularization, day	5.44 (3.14, 9.94)	5.90 (4.21, 6.97)	0.927
*Angiographic features*			
Multivessel disease, (*n*, %)	16 (64.0)	23 (76.7)	0.303
Culprit artery, (*n*, %)			0.098
LAD	12 (48.0)	8 (26.7)	
LCX	6 (24.0)	5 (16.7)	
RCA	7 (28.0)	17 (56.7)	
*Auxiliary examination*			
LVEF−TTE, %	52.24 ± 10.00	48.71 ± 11.78	0.249
*PET/MR parameters*			
Days from onset to scan, day	61 [38, 96]	59 [32, 91]	0.393
LVEF−PET/MR, %	49.39 ± 15.19	49.18 ± 17.71	0.963
GLS	−12.26 (−13.78, −8.17)	−10.44 (−13.75, −7.64)	0.684
GRS	26.03 (14.74, 31.84)	21.80 (13.65, 29.42)	0.493
GCS	−14.08 (−18.35, −10.82)	−15.16 (−18.16, −10.26)	0.761
T1, ms	1268.20 ± 80.81	1239.18 ± 82.55	0.212
T2, ms	51.87 ± 5.03	55.45 ± 4.54	**0.010**
ECV	41.27 (36.64, 45.93)	38.99 (35.14, 45.16)	0.619
*Culprit artery rest MBF, mL/min/g*			
LAD	1.04 ± 0.49	0.81 ± 0.31	0.278
LCX	0.96 ± 0.21	0.83 ± 0.13	0.598
RCA	0.93 ± 0.41	0.80 ± 0.26	0.429
*Biomarkers*			
Peak CKMB, ng/mL	258.50 (6.12, 500.00)	143.00 (16.60, 500.00)	0.874
Peak cTnI, ng/mL	32.90 (0.96, 50.00)	12.40 (1.04, 50.00)	0.543
NT‐proBNP, pg/mL	388.00 (108.35, 4009.50)	352.50 (182.50, 860.25)	0.535
*Laboratory tests*			
WBC, 10^9^/L	8.47 (6.68, 11.11)	7.96 (6.08, 9.26)	0.319
LDL‐C, mmol/L	2.76 (1.90, 3.81)	2.64 (1.94, 3.08)	0.327
HbA1c, %	6.90 (5.80, 7.90)	6.55 (5.60, 8.15)	0.544
hs‐CRP, mg/L	12.03 (6.16, 28.39)	4.26 (2.26, 10.31)	**0.020**
IL‐6, pg/mL	15.07 (8.51, 65.72)	18.40 (8.53, 37.15)	0.957
*Myocardial viability, % LV*			
Hypoperfused myocardium	15.00 (9.00, 25.50)		
LGE	18.00 (12.00, 25.80)	23.00 (10.00, 32.50)	0.483

*Note:* Values are presented as mean ± SD, n (%) or median (IQR).

Abbreviations: AMI = acute myocardial infarction, BMI = body mass index, CKMB = creatine kinase MB, cTnI = cardiac troponin I, ECV = extracellular volume, GCS = global circumferential strain, GLS = global longitudinal strain, GRS = global radial strain, HbA1c = glycosylated hemoglobin, hs‐CRP = high‐sensitivity C‐reactive protein, IL‐6 = interleukin‐6, IQR = interquartile range, LAD = left anterior descending, LCX = left circumflex, LDL‐C = low density lipoprotein cholesterol, LGE = late gadolinium enhancement, LVEF = left ventricular ejection fraction, MBF = myocardial blood flow, NSTEMI = non‐ ST‐segment elevation myocardial infarction, NT‐proBNP = N‐terminal pro‐B‐type natriuretic peptide, PET/MR = positron emission tomography/magnetic resonance, RCA = right coronary artery, SD = standard deviation, STEMI = ST‐segment elevation myocardial infarction, TTE = transthoracic echocardiography, UA = unstable angina, WBC = white blood cell.

Multivariable logistic regression analysis was performed to identify factors associated with the presence of hypoperfused myocardium. After adjustment for age, sex, AMI type, overweight, current smoking, hypertension, diabetes mellitus, multivessel disease, and LVEF measured by PET/MR, T2 was inversely associated with the presence of hypoperfused myocardium (OR = 0.690, 95% CI: 0.508–0.937, *p* = 0.018), whereas hs‐CRP was positively associated with the presence of hypoperfused myocardium (OR = 1.132, 95% CI: 1.031–1.244, *p* = 0.009) (Table 2).

**Table 2:** Multivariable logistic regression of the presence of hypoperfused myocardium.

Variates	OR	95% CI	*p* value
Age	1.012	0.916−1.118	0.812
Female	0.006	0.000−1.224	0.107
AMI type—STEMI	10.806	0.945−123.563	0.066
Overweight	0.488	0.023−10.521	0.647
Current smoking	0.310	0.035−2.713	0.290
Hypertension	1.619	0.224−11.718	0.633
Diabetes mellitus	0.161	0.011−2.410	0.186
Multivessel disease	2.949	0.217−40.052	0.416
LVEF—PET/MR	0.944	0.863−1.034	0.214
T2	0.690	0.508−0.937	0.018
hs‐CRP	1.132	1.031−1.244	0.009

Abbreviations: CI = confidence interval, hs‐CRP = high‐sensitivity C‐reactive protein, LVEF = left ventricular ejection fraction, OR = odds ratio, PET/MR = positron emission tomography/magnetic resonance, STEMI = ST‐segment elevation myocardial infarction.

To minimize the potential influence of heterogeneity between STEMI and NSTEMI patients, baseline characteristics were compared (Supporting Information S1: Table 1). No significant differences were found in demographics or comorbidities, with STEMI patients exhibiting larger infarct size, higher peak myocardial enzyme levels, and higher ECV—consistent with the greater ischemic burden typical of STEMI. Besides, AMI type was adjusted for in the multivariable logistic regression model (Table 2). Subgroup analyses were further performed according to AMI type, showing that the associations of T2 and hs‐CRP with the presence of hypoperfused myocardium were generally consistent in both STEMI and NSTEMI patients. No significant interaction was observed between AMI type and T2 or hs‐CRP, with P for interaction values of 0.264 and 0.683, respectively (Supporting Information S1: Table 2).

### 3.3 Changes in PET/MR and Biochemical Parameters From Baseline to Follow‐Up

**Figure 2:**
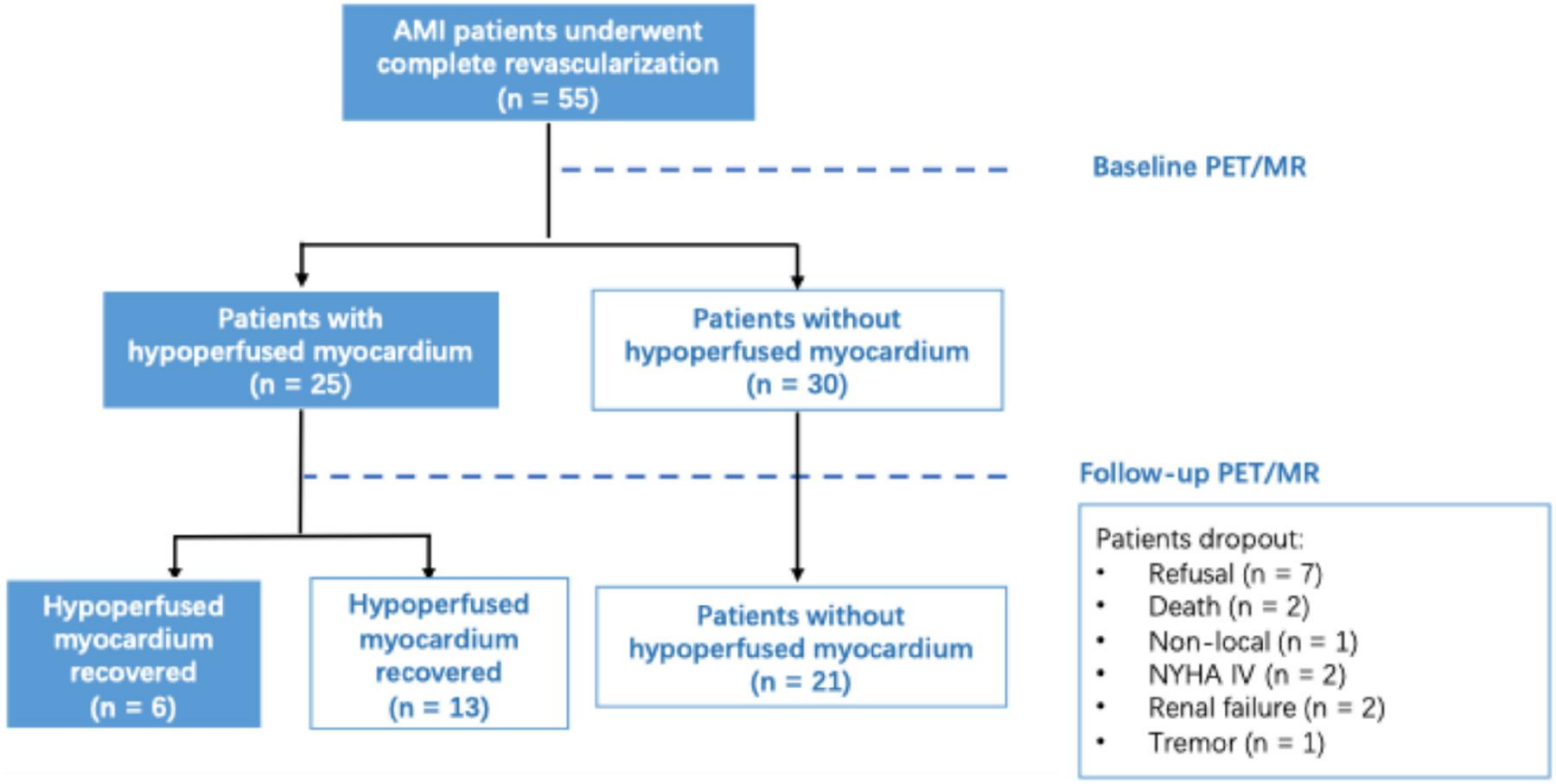
Patient flowchart. Patient flowchart of the study. Fifty‐five AMI patients were prospectively enrolled after complete revascularization from October 2020 to July 2021. Baseline and follow‐up PET/MR scans were scheduled at 2 months and 1 year after AMI, and dropouts were listed in detail. AMI = acute myocardial infarction; NYHA = New York Heart Association; PET/MR = positron emission tomography/magnetic resonance.

Forty patients completed follow‐up PET/MR imaging at 401 (375, 428) days after admission, and their clinical characteristics were presented in Supporting Information S1: Table 3 and 4, which were similar to those of the whole cohort. The reasons for dropouts are listed in detail in the flowchart (Figure 2).

Patients in Group A showed LV ejection fraction (LVEF) improvement (56.39 ± 9.76 vs. 54.58 ± 10.39, *p* = 0.016) at follow‐up, while those in Group B did not. A significant reduction of IL‐6 and white blood cell (WBC) count was observed in both groups. Variations of PET/MR and biochemical parameters between baseline and follow‐up are listed in Table 3.

**Table 3:** Variation of parameters between baseline and follow‐up scan.

Variables	Hypoperfused myocardium (+) (*n* = 19)			Hypoperfused myocardium (–) (*n* = 21)		
	Baseline	Follow‐up	*p* value	Baseline	Follow‐up	*p* value
PET/MR						
LVEDV, mL	136.98 (105.50, 156.02)	135.25 (105.75, 145.95)	0.831	143.47 (119.34, 182.29)	154.70 (117.14, 168.47)	0.643
LVESV, mL	60.27 (36.61, 69.55)	54.59 (37.13, 71.56)	0.224	68.40 (39.90, 108.29)	71.41 (46.64, 98.52)	0.819
LVEF, %	54.58 ± 10.39	56.39 ± 9.76	**0.016**	50.79 ± 16.95	51.63 ± 13.09	0.615
Myocardium volume, mL	89.71 ± 27.14	87.67 ± 25.31	0.341	104.62 ± 26.37	99.62 ± 17.75	0.148
Myocardium mass, g	98.74 ± 28.24	94.58 ± 25.58	0.068	107.15 ± 21.07	106.48 ± 18.72	0.969
GLS	−12.32 ± 2.48	−12.82 ± 3.19	0.306	−10.97 ± 4.22	−11.38 ± 3.19	0.638
GRS	26.95 ± 11.30	25.87 ± 8.96	0.542	23.05 ± 8.59	23.18 ± 10.41	0.949
GCS	−15.94 ± 3.57	−15.67 ± 3.41	0.559	−15.29 ± 4.54	−15.28 ± 3.99	0.987
T1, ms	1246.16 ± 68.28	1123.89 ± 55.48	**< 0.001**	1241.13 ± 69.39	1149.81 ± 60.64	**< 0.001**
T2, ms	51.39 ± 5.05	51.09 ± 5.60	0.794	55.90 ± 4.38	53.46 ± 4.67	0.064
ECV	40.78 ± 7.59	39.94 ± 6.59	0.619	39.62 ± 5.26	41.80 ± 7.92	0.184
Hypoperfused myocardium, % LV	15.00 (9.00, 24.00)	12.00 (6.00, 18.00)	**0.013**			
LGE, % LV	17.33 (12.86, 25.60)	13.93 (9.42, 21.76)	**0.044**	23.00 (10.00, 29.50)	18.70 (9.98, 24.71)	**0.037**
Laboratory tests						
hs‐CRP, mg/L	11.78 (5.40, 32.14)	0.82 (0.33, 2.44)	**0.004**	4.66 (2.26, 15.58)	0.72 (0.25, 8.34)	0.266
IL‐6, pg/mL	14.60 (6.90, 66.64)	2.26 (1.52, 3.42)	**0.001**	25.29 (12.26, 43.39)	3.80 (1.65, 8.07)	**0.017**
HbA1c, %	6.50 (5.80, 8.03)	6.20 (5.80, 7.20)	0.413	6.35 (5.60, 7.35)	6.40 (5.65, 7.35)	0.964
WBC, 10^^^9/L	8.47 (7.32, 11.14)	6.89 (5.80, 7.58)	**0.010**	7.82 (5.90, 8.97)	6.11 (5.29, 7.20)	**0.029**

*Note:* Values are presented as mean ± SD, *n* (%) or median (IQR).

Abbreviations: ECV = extracellular volume, GCS = global circumferential strain, GLS = global longitudinal strain, GRS = global radial strain, HbA1c = glycosylated hemoglobin, hs‐CRP = high‐sensitivity C‐reactive protein, IL‐6 = interleukin‐6, IQR = interquartile range, LGE = late gadolinium enhancement, LVEDV = left ventricular end‐diastolic volume, LVEF = left ventricular ejection fraction, LVESV = left ventricular end‐systolic volume, PET/MR = positron emission tomography/magnetic resonance, SD = standard deviation, STEMI = ST‐segment elevation myocardial infarction, WBC = white blood cell.

### 3.4 Characteristics of Patients With the Recovery of Hypoperfused Myocardium

**Figure 3:**
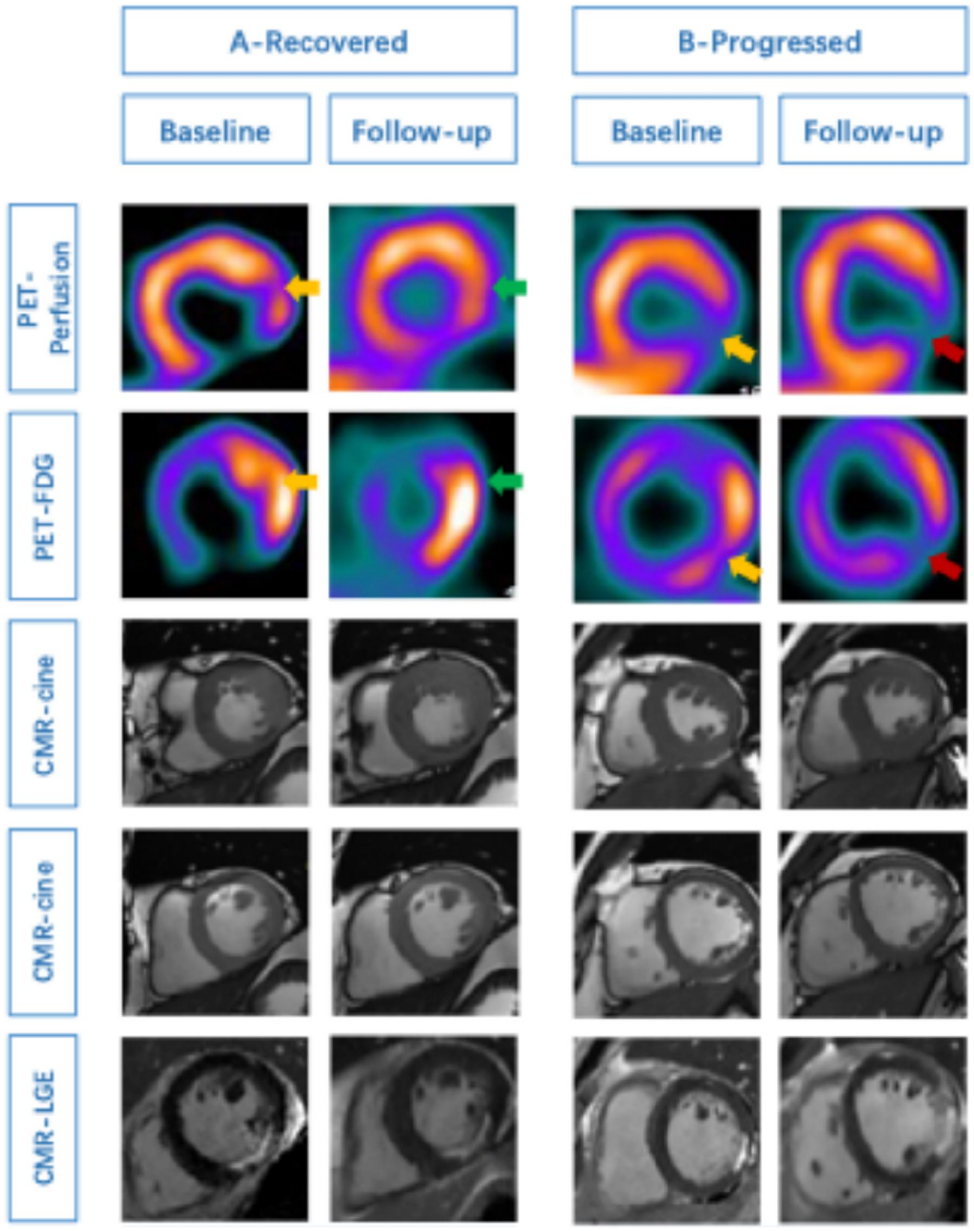
Representative PET/MR image of 2 separate patients. (A) Short‐axis images obtained from 2‐month and 1‐year scans of a 49‐year‐old male who initially presented with inferior STEMI and underwent PCI to his RCA. Hypoperfused myocardium was observed in the mid inferolateral wall at baseline scan (yellow arrow), and recovered at follow‐up (green arrow), with evidence of ameliorated perfusion and improved segmental wall movement. (B) Short‐axis images obtained from 2‐month and 1‐year scans of a 43‐year‐old male who also initially presented with inferior STEMI and underwent PCI to his LCX. Hypoperfused myocardium was observed in the basal inferolateral wall at baseline scan (yellow arrow), and progressed to scar at follow‐up (red arrow), showing diminished metabolism and no significant improvement in segmental wall movement. FDG = fluorodeoxyglucose; GLS = global longitudinal strain; hs‐CRP = high‐sensitivity C‐reactive protein. LCX = left circumflex; LGE = late gadolinium enhancement; PCI = percutaneous coronary intervention; PET/MR = positron emission tomography/magnetic resonance; RCA = right coronary artery; STEMI = ST‐segment elevation myocardial infarction.

The recovery of hypoperfused myocardium was detected in 6 of the 19 patients in Group A (Figure 3). In patients with the recovery of hypoperfused myocardium, there were more females (66.7% vs. 15.4%, *p* = 0.025), fewer diabetics (0.0% vs. 46.2%, *p* = 0.044), and fewer overweight (50.0% vs. 92.3%, *p* = 0.035) than patients without. Besides, patients with the recovery of hypoperfused myocardium had higher GLS at baseline and follow‐up than patients without [−14.50 ± 1.94 vs. −11.31 ± 2.05, *p* = 0.005, and −15.57 ± 3.20 vs. −11.55 ± 2.34, *p* = 0.006, respectively]. There was no difference in angiographic and procedural parameters or laboratory tests. Detailed findings of comparison between groups are displayed in Table 4.

**Table 4:** Clinical characteristics and PET/MR parameters according to the evolution of hypoperfused myocardium.

Variable	Hypoperfused myocardium recovery (+) (*n* = 6)	Hypoperfused myocardium recovery (−) (*n* = 13)	*p* value
Demographic			
Age, years	59.67 ± 13.11	59.46 ± 10.15	0.971
Male, (*n*, %)	2 (33.3)	11 (84.6)	**0.025**
BMI, kg/m^2^	23.68 ± 3.43	25.65 ± 2.26	0.152
Type of AMI			
STEMI, *n* (%)	3 (50, 0)	7 (53.8)	0.876
Cardiovascular risk factors			
Hypertension, (*n*, %)	3 (50.0)	10 (76.9)	0.320
Hyperlipidemia, (*n*, %)	1 (16.7)	2 (15.4)	0.943
Diabetes mellitus, (*n*, %)	0 (0.0)	6 (46.2)	**0.044**
Previous MI, (*n*, %)	1 (16.7)	1 (7.7)	0.554
Smoking, (*n*, %)	2 (33.3)	8 (61.5)	0.350
Overweight or obesity, (*n*, %)	3 (50.0)	12 (92.3)	**0.035**
Signs			
SBP, mmHg	121.00 ± 6.33	126.38 ± 24.24	0.604
DBP, mmHg	73.00 ± 17.09	71.69 ± 14.17	0.863
HR, bpm	81.67 ± 10.91	84.08 ± 15.97	0.743
Auxiliary examination			
LVEF−TTE, %	55.33 ± 8.02	55.31 ± 7.89	0.995
Cardiac biomarkers			
Peak CKMB, ng/mL	191.0 (80.1, 465.5)	322.0 (85.4, 483.5)	0.724
Peak cTnI, ng/mL	33.50 (4.62, 46.75)	41.15 (10.09, 50.00)	0.724
NT‐proBNP, pg/mL	108 (54, 2150)	190 (121, 3143)	0.272
Laboratory tests			
WBC, 10^9^/L	8.53 ± 1.76	9.53 ± 3.32	0.499
Hgb, g/L	135.17 ± 23.91	137.08 ± 16.37	0.840
Creatinine, *μ*mol/L	59.50 (38.50, 86.00)	62.00 (54.00, 75.00)	0.831
LDL‐C, mmol/L	3.47 (1.59, 4.00)	2.76 (1.94, 3.69)	0.639
HbA1c, %	6.83 ± 2.18	7.02 ± 1.67	0.846
hs‐CRP, mg/L	11.50 (1.10, 11.78)	9.71 (5.65, 42.00)	0.630
IL‐6, pg/mL	13.83 (5.88, 52.49)	14.60 (6.90, 70.97)	0.710
Length of stay, day	10.50 (6.25, 21.75)	8.00 (6.50, 11.50)	0.467
Angiography and reperfusion			
Multivessel disease (*n*, %)	4 (66.7)	8 (61.5)	0.998
Culprit artery (*n*, %)			
LAD	4 (66.7)	5 (38.5)	0.626
LCX	1 (16.7)	6 (46.2)	
RCA	1 (16.7)	2 (15.4)	
PCI (*n*, %)	6 (100)	10 (76.9)	0.440
CABG (*n*, %)	0 (0)	1 (7.7)	
Number of stents implanted	1.50 (1.00, 2.50)	1.00 (0.00, 2.00)	0.127
PET/MR parameters			
Morphology and structure			
LVEDV, mL	94.92 (81.58, 148.10)	140.66 (117.57, 162.72)	0.106
LVESV, mL	34.86 (28.70, 67.79)	64.91 (55.04, 75.79)	0.058
Myocardium volume, mL	68.18 (59.72, 92.86)	97.30 (69.63, 111.86)	0.179
Myocardium mass, g	65.83 (62.86, 107.06)	109.10 (81.15, 127.68)	0.058
Movement function			
LVEF ‐ PET/MR, %	61.11 ± 10.08	51.57 ± 9.41	0.081
SV, mL	69.32 (46.33, 80.32)	71.63 (58.53, 87.19)	0.701
CO, L	4.48 (3.17, 5.32)	5.15 (3.76, 5.66)	0.323
Strain			
GLS	−14.50 ± 1.94	−11.31 ± 2.05	**0.005**
GRS	32.51 ± 15.05	24.39 ± 8.64	0.416
GCS	−18.00 ± 3.47	−14.99 ± 3.32	0.127
T1, ms	1227.38 ± 76.22	1255.55 ± 65.39	0.426
T2, ms	48.39 ± 2.80	52.90 ± 5.33	0.072
ECV	38.56 ± 8.30	41.89 ± 7.32	0.750
Hypoperfused myocardium, % LV	14.89 (8.00, 21.50)	15.02 (11.40, 25.92)	0.151
LGE, % LV	11.27 (6.44, 21.24)	12.36 (9.68, 26.80)	0.180

*Note:* Values are presented as mean ± SD, *n* (%) or median (interquartile range).

Abbreviations: AMI = acute myocardial infarction, CABG = coronary artery bypass graft, CKMB = creatine kinase MB, CO = cardiac output, cTnI = cardiac troponin I, DBP = diastolic blood pressure, ECV = extracellular volume, GCS = global circumferential strain, GLS = global longitudinal strain, GRS = global radial strain, HbA1c = glycosylated hemoglobin, Hgb = hemoglobulin, hs‐CRP = high‐sensitivity C‐reactive protein, HR = heart rate, IL‐6 = interleukin‐6, LAD = left anterior descending, LCX = left circumflex, LGE = late gadolinium enhancement, LVEDV = left ventricular end‐diastolic volume, LVEF = left ventricular ejection fraction, LVESV = left ventricular end‐systolic volume, MI = myocardial infarction, NT‐proBNP = N‐terminal pro‐B‐type natriuretic peptide, PCI = percutaneous coronary intervention, RCA = right coronary artery, SBP = systolic blood pressure, STEMI = ST‐segment elevation myocardial infarction, SV = stroke volume, TTE = transthoracic echocardiography.

## 4 Discussion

This study described myocardial hypoperfusion, dynamic, and association with LV systolic function visualized by PET/MR imaging at 2 months and 1 year after AMI. The main results are as follows: (1) nearly half of the AMI patients showed hypoperfused myocardium at 2 months after complete revascularization; (2) patients with hypoperfused myocardium had higher hs‐CRP and lower T2 value; (3) patients with hypoperfused myocardium had improved LVEF at 1‐year follow‐up, and (4) the recovery of hypoperfused myocardium was more frequently seen in female patients without diabetes or overweight, and with higher GLS levels.

### 4.1 Hypoperfused Myocardium in Revascularized AMI Patients

Coronary revascularization restores epicardial flow but does not necessarily normalize myocardial perfusion [17]. Perfusion defects can be detected by angiography, contrast echocardiography, nuclear imaging, and CMR [18]. Experimental studies have also shown that, despite restored epicardial patency after coronary occlusion, portions of the subendocardial microvasculature may remain unperfused [19]. Consistently, our study found hypoperfused myocardium in a substantial proportion of AMI patients months after complete revascularization, suggesting that impaired myocardial perfusion may persist beyond the acute phase.

### 4.2 Underlying Mechanisms of Hypoperfusion

Impaired tissue reperfusion may arise from myocyte swelling, endothelial injury, distal embolization, and ischemia/reperfusion injury [20]. MVO, observed in 57% of reperfused STEMI patients [21], is strongly associated with long‐term iron deposition within the infarct core [22]. In this study, myocardial hypoperfusion is accompanied by a lower T2 value. T2 and T2* mapping techniques detect myocardial iron deposition via the paramagnetism of deoxyhemoglobin, and T2 values are negatively correlated with iron deposition [23]. A lower T2 value could be the sequelae of MVO and intramyocardial hemorrhage, which is concordant with the retention of hypoperfusion.

Meanwhile, inflammation is a key pathogenic driver of impaired tissue perfusion, with inflammatory markers exerting dose‐dependent effects on microcirculatory dysfunction [24]. Higher hs‐CRP levels were seen in patients with hypoperfused myocardium in this study, consistent with previous evidence linking heightened inflammation to adverse outcomes. Given that anti‐inflammatory therapy has been shown in randomized trials to reduce adverse cardiac events [25, 26], our findings suggest that hypoperfused myocardium accompanied by elevated inflammatory activity may represent a potential therapeutic target.

### 4.3 Hypoperfused Myocardium Dynamic and LV Systolic Function

It is noteworthy that the dynamic evolution of hypoperfused myocardium occurred beyond the first months postinfarction. At 1‐year follow‐up, a modest but significant increase in LVEF was observed in patients in Group A, and approximately 1/3 of patients had their hypoperfused myocardium recovered to normal.

Different imaging modalities assess dysfunctional myocardium from distinct pathophysiological perspectives, including contractile reserve by dobutamine‐stressed echocardiography (DSE), membrane integrity by single‐photon emission computed tomography (SPECT), glucose metabolism by PET, and infarct extent by CMR‐LGE. Such heterogeneity may lead to discordant findings and obscure interpretation [15]. Hybrid PET/MR enables integrated evaluation of myocardial perfusion, metabolism, morphology, and contractility with superior co‐registration, motion correction, and reduced radiation exposure [27]. The current study visually assessed myocardial perfusion and contractile function, and suggested a potential association between recovery of hypoperfused myocardium and improvement in LV systolic function.

Different patterns of hypoperfused myocardium evolution were observed in this study. Around 1/3 of the patients experienced reversal, while the rest showed no significant change or even progressed to scar. The explicit determinants were not clear, but some specific clinical features like female gender, non‐diabetic or overweight, and better strain were found in patients with recovered hypoperfused myocardium, suggesting those characteristics could possibly be involved in the dynamics of hypoperfused myocardium. Moreover, previous literature described a spectrum of ischemia‐jeopardized myocardium including stunning and hibernation, characterized as viable but dysfunctional, portions of which might benefit from interventions, but the trajectory, time course, and potential mechanisms remained unclear [15]. We speculate that cardiac dysfunction due to myocardial hypoperfusion may be a potential explanation for the lasting stunning or hibernation even after restoration of the epicardial blood flow.

## 5 Study Limitations

There are some limitations in this study. First, the sample size in this study is relatively small, and patients were not consecutively enrolled. There were dropouts during follow‐up because of refusal or disease progression, which might lead to selection bias. Second, there are variations in the duration from onset to the baseline PET/MR scan among patients, but no significant difference between groups was observed. Third, multivariable regression analysis for positive recovery of hypoperfused myocardium was not performed because the limited number of outcome events would have substantially reduced statistical power and increased the risk of unstable estimates and model overfitting. Fourth, formal inter‐observer and intra‐observer reproducibility analyses were not performed; therefore, the lack of quantitative reproducibility assessment remains a methodological limitation, particularly in evaluating the robustness of hypoperfused myocardium quantification. Finally, current research findings indicate that hypoperfused myocardium might be associated with recovery in LV systolic function, but whether or not this could bring up survival benefits remains unclear. Further validation in larger, multicenter studies is warranted.

## 6 Conclusion

Hypoperfused myocardium is observed in nearly half of the AMI patients 1 year after complete revascularization, which might be associated with a higher level of inflammation and microvascular dysfunction. Recovery of hypoperfusion may be associated with improved cardiac systolic function.

## Author Contributions

Conception and design: Jing Li, Jie Lu. Acquisition, analysis, and interpretation of data: Jin Si, Chong Zheng, Jinghao Sun, Keling Xiao, Yadong Cui, Bixiao Cui, Ming Yi, Lijie Sun, Haoyu Zhang, Shanshan Gu. Drafting and revising of the work: Jin Si, Chong Zheng, Jinghao Sun, Jie Lu, Jing Li. Approval of the manuscript submitted: all authors.

## Ethics Statement

This study was performed in line with the principles of the Declaration of Helsinki. Approval was granted by the Ethics Committee of Xuanwu Hospital Capital Medical University (Clinical trial [2022] No.169), and written informed consent was obtained from all patients.

## Consent

Written informed consent for publication was obtained from all patients.

## Conflicts of Interest

The authors declare no conflicts of onterest.

## Supporting information


Supporting File

## Data Availability

The corresponding author has full access to all the data in the study and takes responsibility for its integrity and data analysis.
