# Supplementary material for: The Association between Hypoperfused Myocardium and Systolic Function in Patients With AMI Undergoing Complete Revascularization
Source: Clin Cardiol. 2026 Aug 6;49(8):e70437. doi: 10.1002/clc.70437 (PMC13446020; doi:10.1002/clc.70437)
Supplement: Supplementary file 1 [file CLC-49-e70437-s001.docx]

**Table S1. Baseline characteristics of patients stratified by AMI type**

| Variable | STEMI  (n = 28) | NSTEMI  (n = 27) | P-value |
| --- | --- | --- | --- |
| Demographics |  |  |  |
| Age, years | 58.64 ± 12.46 | 62.26 ± 10.26 | 0.246 |
| Male, (n, %) | 21 (75.0) | 23 (85.2) | 0.503 |
| Body mass index, kg/m^2^ | 24.98 ± 2.58 | 25.41 ± 2.80 | 0.550 |
| Cardiovascular risk factors |  |  |  |
| Hypertension, (n, %) | 14 (50.0) | 18 (66.7) | 0.277 |
| Diabetes mellitus, (n, %) | 14 (50.0) | 8 (29.6) | 0.171 |
| Smoking, (n, %) | 17 (60.7) | 16 (59.3) | >0.999 |
| Overweight or obesity, (n, %) | 19 (67.9) | 21 (77.8) | 0.547 |
| Angiographic features |  |  |  |
| Multivessel disease, (n, %) | 20 (71.4) | 20 (74.1) | >0.999 |
| Culprit artery, (n, %) |  |  | >0.999 |
| LAD | 10 (35.7) | 9 (33.3) |  |
| LCX | 6 (21.4) | 6 (22.2) |  |
| RCA | 12 (42.9) | 12 (44.4) |  |
| PET/MR parameters |  |  |  |
| LVEF - PET/MR, % | 46.59 ± 14.55 | 52.05 ± 18.09 | 0.222 |
| GLS | -9.57 (-13.55, -6.73) | -10.52 (-13.38, -8.83) | 0.142 |
| GRS | 20.95 (13.65, 27.39) | 25.59 (20.42, 29.53) | 0.560 |
| GCS | -14.30 (-17.69, -10.26) | -14.66 (-18.51, -13.05) | 0.460 |
| T1, ms | 1270.90 ± 83.22 | 1232.00 ± 78.14 | 0.088 |
| T2, ms | 54.34 ± 4.40 | 53.53 ± 5.65 | 0.569 |
| ECV | 44.82 (36.82, 46.65) | 35.26 (30.27, 39.91) | **0.001** |
| Biomarkers |  |  |  |
| Peak CKMB, ng/mL | 434.00 (149.00, 500.00) | 58.95 (27.55, 114.35) | **<0.001** |
| Peak cTnI, ng/mL | 44.30 (10.08, 50.00) | 5.22 (1.19, 19.10) | **<0.001** |
| NT-proBNP, pg/mL | 388.00 (96.75, 2887.00) | 1008.00 (201.93, 2508.75) | 0.470 |
| Laboratory tests |  |  |  |
| WBC, 10^9^/L | 9.96 ± 3.47 | 7.40 ± 2.21 | **0.002** |
| LDL-C, mmol/L | 2.96 ± 0.80 | 2.36 ± 0.88 | **0.011** |
| HbA1c, % | 7.22 ± 2.11 | 6.54 ± 1.08 | 0.153 |
| hs-CRP, mg/L | 9.20 (3.58, 19.04) | 4.20 (1.32, 17.10) | 0.517 |
| IL-6, pg/mL | 19.92 (12.27, 54.02) | 6.15 (3.29, 37.82) | 0.060 |
| Myocardial viability |  |  |  |
| Hypoperfused myocardium, % LV | 0.00 (0.00, 12.00) | 0.00 (0.00, 9.75) | 0.541 |
| LGE, % LV | 25.00 (20.85, 32.50) | 11.35 (7.02, 21.50) | **0.012** |

Values are presented as mean ± SD, n (%) or median (interquartile range).

**Abbreviations**: AMI = acute myocardial infarction; BMI = body mass index; CKMB = creatine kinase MB; cTnI = cardiac troponin I; ECV = extracellular volume; GCS = global circumferential strain; GLS = global longitudinal strain; GRS = global radial strain; HbA1c = glycosylated hemoglobin; hs-CRP = high-sensitivity C-reactive protein; IL-6 = interleukin-6; LAD = left anterior descending; LCX = left circumflex; LDL-C = low density lipoprotein cholesterol; LGE = late gadolinium enhancement; LVEF = left ventricular ejection fraction; NT-proBNP = N-terminal pro-B-type natriuretic peptide; PET/MR = positron emission tomography/magnetic resonance; RCA = right coronary artery; SD = standard deviation; WBC = white blood cell.

**Table S2. Subgroup analyses of the associations of T2 and hs-CRP with hypoperfused myocardium stratified by AMI type**

| Variable | AMI type | Adjusted OR | 95% CI | P-value | P for interaction |
| --- | --- | --- | --- | --- | --- |
| T2 | STEMI | 0.637 | 0.415-0.977 | 0.039 | 0.264 |
| T2 | NSTEMI | 0.730 | 0.518-0.942 | 0.048 |  |
| hs-CRP | STEMI | 1.079 | 1.013-1.168 | 0.019 | 0.683 |
| hs-CRP | NSTEMI | 1.146 | 1.002-1.242 | 0.046 |  |

ORs were calculated using multivariable logistic regression models adjusted for age, sex, overweight, current smoking, hypertension, diabetes mellitus, multivessel disease, and LVEF measured by PET/MR. T2 and hs-CRP were analyzed as continuous variables. P for interaction was derived from the interaction term between AMI type and each centered continuous variable.

**Abbreviations**: AMI = acute myocardial infarction; CI = confidence interval; hs-CRP = high-sensitivity C-reactive protein; LVEF = left ventricular ejection fraction; NSTEMI = non-ST-segment elevation myocardial infarction; OR = odds ratio; PET/MR = positron emission tomography/magnetic resonance; STEMI = ST-segment elevation myocardial infarction.

**Table S3. Baseline characteristics of patients stratified by follow-up status (lost to follow-up vs. completed two scans)**

| Variable | Patients lost to follow-up  (n = 15) | Patients completed two scans  (n = 40) | P-value |
| --- | --- | --- | --- |
| Demographics |  |  |  |
| Age, years | 59.93 ± 14.97 | 60.60 ± 10.08 | 0.850 |
| Male, (n, %) | 13 (86.7) | 31 (77.5) | 0.708 |
| Body mass index, kg/m^2^ | 25.50 ± 3.29 | 25.08 ± 2.45 | 0.611 |
| Type of AMI |  |  |  |
| STEMI, n (%) | 8 (53.3) | 20 (50.0) | >0.999 |
| Cardiovascular risk factors |  |  |  |
| Hypertension, (n, %) | 10 (66.7) | 22 (55.0) | 0.545 |
| Diabetes mellitus, (n, %) | 10 (66.7) | 12 (30.0) | **0.028** |
| Smoking, (n, %) | 10 (66.7) | 23 (57.5) | 0.758 |
| Overweight or obesity, (n, %) | 10 (66.7) | 30 (75.0) | 0.735 |
| Angiographic features |  |  |  |
| Multivessel disease, (n, %) | 13 (86.7) | 27 (67.5) | 0.192 |
| Culprit artery, (n, %) |  |  | 0.424 |
| LAD | 5 (33.3) | 14 (35.0) |  |
| LCX | 5 (33.3) | 7 (17.5) |  |
| RCA | 5 (33.3) | 19 (47.5) |  |
| PET/MR parameters |  |  |  |
| LVEF - PET/MR, % | 48.42 ± 19.24 | 52.59 ± 14.17 | 0.230 |
| GLS | -6.87 (-10.37, -5.45) | -10.98 (-14.05, -8.16) | **0.004** |
| GRS | 15.48 (13.57, 24.10) | 23.69 (18.89, 29.96) | **0.012** |
| GCS | -12.98 (-16.45, -10.94) | -15.16 (-18.33, -10.06) | **0.031** |
| T1, ms | 1275.46 ± 115.34 | 1243.45 ± 68.02 | 0.359 |
| T2, ms | 54.28 ± 4.79 | 53.82 ± 5.16 | 0.779 |
| ECV | 44.44 (37.21, 52.16) | 41.21 (35.21, 45.91) | 0.207 |
| Biomarkers |  |  |  |
| Peak CKMB, ng/mL | 389.5 (46.7, 500.0) | 269.0 (79.4, 500.0) | 0.902 |
| Peak cTnI, ng/mL | 33.80 (3.64, 50.00) | 32.30 (7.15, 50.00) | 0.821 |
| NT-proBNP, pg/mL | 861.5 (137.5, 3868.8) | 371.0 (93.7, 2310.0) | 0.128 |
| Laboratory tests |  |  |  |
| WBC, 10^9^/L | 9.43 ± 4.12 | 8.43 ± 2.74 | 0.299 |
| LDL-C, mmol/L | 2.47 ± 0.86 | 2.74 ± 0.90 | 0.325 |
| HbA1c, % | 7.47 ± 2.20 | 6.78 ± 1.65 | 0.268 |
| hs-CRP, mg/L | 6.70 (3.23, 11.47) | 9.20 (2.80, 22.50) | 0.506 |
| IL-6, pg/mL | 20.49 (11.54, 46.06) | 19.27 (8.52, 56.78) | 0.554 |
| Myocardial viability |  |  |  |
| Hypoperfused myocardium, % LV | 0.0 (0.0, 10.5) | 0.0 (0.0, 12.0) | 0.664 |
| LGE, % LV | 25.5 (22.0, 39.5) | 23.0 (13.7, 27.0) | 0.131 |

Values are presented as mean ± SD, n (%) or median (interquartile range).

**Abbreviations**: AMI = acute myocardial infarction; BMI = body mass index; CKMB = creatine kinase MB; cTnI = cardiac troponin I; ECV = extracellular volume; GCS = global circumferential strain; GLS = global longitudinal strain; GRS = global radial strain; HbA1c = glycosylated hemoglobin; hs-CRP = high-sensitivity C-reactive protein; IL-6 = interleukin-6; LAD = left anterior descending; LCX = left circumflex; LDL-C = low density lipoprotein cholesterol; LGE = late gadolinium enhancement; LVEF = left ventricular ejection fraction; NT-proBNP = N-terminal pro-B-type natriuretic peptide; PET/MR = positron emission tomography/magnetic resonance; RCA = right coronary artery; SD = standard deviation; WBC = white blood cell.

**Table S4. Baseline characteristics of patients who completed two scans stratified by the presence of hypoperfused myocardium**

| Variable | Hypoperfused myocardium +  (n = 19) | Hypoperfused myocardium -  (n = 21) | P-value |
| --- | --- | --- | --- |
| Demographics |  |  |  |
| Age, years | 59.53 ± 10.79 | 61.57 ± 9.55 | 0.529 |
| Male, (n, %) | 13 (68.4) | 18 (85.7) | 0.265 |
| BMI, kg/m^2^ | 25.03 ± 2.75 | 25.12 ± 2.21 | 0.903 |
| Type of AMI |  |  |  |
| STEMI, n (%) | 10 (52.6) | 10 (47.6) | 0.989 |
| Cardiovascular risk factors |  |  |  |
| Hypertension, (n, %) | 13 (68.4) | 9 (42.9) | 0.125 |
| Diabetes mellitus, (n, %) | 6 (31.6) | 6 (28.6) | 0.836 |
| Smoking, (n, %) | 10 (52.6) | 13 (61.9) | 0.750 |
| Overweight or obesity, (n, %) | 15 (78.9) | 15 (71.4) | 0.721 |
| Angiographic features |  |  |  |
| Multivessel disease, (n, %) | 12 (63.2) | 15 (71.4) | 0.738 |
| Culprit artery, (n, %) |  |  |  |
| LAD | 7 (36.8) | 7 (33.3) | 0.108 |
| LCX | 6 (31.6) | 3 (14.3) |  |
| RCA | 6 (31.6) | 11 (52.4) |  |
| Auxiliary examination |  |  |  |
| LVEF - TTE, % | 55.32 ± 7.70 | 50.85 ± 11.31 | 0.160 |
| PET/MR parameters |  |  |  |
| LVEF - PET/MR, % | 54.58 ± 10.39 | 50.79 ± 16.95 | 0.406 |
| GLS | -12.79 (-14.00, -10.55) | -11.18 (-14.56, -8.40) | 0.478 |
| GRS | 26.29 (17.81, 33.71) | 23.13 (16.27, 30.56) | 0.380 |
| GCS | -15.80 (-18.37, -13.46) | -15.91 (-19.43, -10.88) | 0.771 |
| T1, ms | 1246.16 ± 68.28 | 1241.13 ± 69.39 | 0.821 |
| T2, ms | 51.39 ± 5.05 | 55.90 ± 4.38 | **0.005** |
| ECV | 40.94 (34.99, 45.93) | 37.98 (35.14, 44.83) | 0.563 |
| Biomarkers |  |  |  |
| Peak CKMB, ng/mL | 269.00 (93.10, 467.00) | 115.90 (15.63, 500.00) | 0.465 |
| Peak cTnI, ng/mL | 33.50 (7.94, 50.00) | 9.66 (0.70, 50.00) | 0.287 |
| NT-proBNP, pg/mL | 153 (90, 3134) | 371 (188, 702) | 0.515 |
| Laboratory tests |  |  |  |
| WBC, 10^9^/L | 9.21 ± 2.90 | 7.72 ± 2.44 | 0.085 |
| LDL-C, mmol/L | 2.88 ± 1.11 | 2.61 ± 0.65 | 0.332 |
| HbA1c, % | 6.96 ± 1.80 | 6.61 ± 1.51 | 0.538 |
| hs-CRP, mg/L | 11.78 (5.40, 32.14) | 4.66 (2.26, 15.58) | **0.018** |
| IL-6, pg/mL | 14.60 (6.90, 66.64) | 25.29 (12.26, 43.39) | 0.550 |
| Myocardial viability |  |  |  |
| Hypoperfused myocardium, % LV | 15 (9, 24) | - | - |
| LGE, % LV | 17 (13, 26) | 23 (10, 29) | 0.568 |

Values are presented as mean ± SD, n (%) or median (interquartile range).

**Abbreviations**: AMI = acute myocardial infarction; BMI = body mass index; CKMB = creatine kinase MB; cTnI = cardiac troponin I; ECV = extracellular volume; GCS = global circumferential strain; GLS = global longitudinal strain; GRS = global radial strain; HbA1c = glycosylated hemoglobin; hs-CRP = high-sensitivity C-reactive protein; IL-6 = interleukin-6; LAD = left anterior descending; LCX = left circumflex; LDL-C = low density lipoprotein cholesterol; LGE = late gadolinium enhancement; LVEF = left ventricular ejection fraction; NT-proBNP = N-terminal pro-B-type natriuretic peptide; PET/MR = positron emission tomography/magnetic resonance; RCA = right coronary artery; SD = standard deviation; WBC = white blood cell.
